# *N*,*N*′-Bis(4-methyl­phen­yl)naphthalene-1,4-dicarboxamide *N*,*N*-dimethyl­acetamide disolvate

**DOI:** 10.1107/S1600536808037422

**Published:** 2008-11-20

**Authors:** Lin-Hai Jing

**Affiliations:** a School of Chemistry and Chemical Engineering, China West Normal University, Nanchong 637002, People’s Republic of China

## Abstract

The title compound, C_26_H_22_N_2_O_2_·2C_4_H_9_NO, crystallizes in an *anti* C=O orientation. The two amide groups are approximately perpendicular to the naphthalene ring system [dihedral angles = 88.89 (1) and 89.08 (1)°]. Each of the dimethyl­acetamide solvent mol­ecules are disordered over two positions, with occupancies of 0.655 (12):0.345 (12) and 0.531 (13):0.469 (13). The crystal packing is stabilized by N—H⋯O and C—H⋯O hydrogen bonds.

## Related literature

For general background to the application of 1,4-naphthalene­dicarboxylic acid derivatives as monomers in the preparation of polymers, see: Fukuzumi *et al.* (1994 ▶); Tsukada *et al.* (1994 ▶). For related structures, see: Jing *et al.* (2006*a* ▶,*b* ▶).
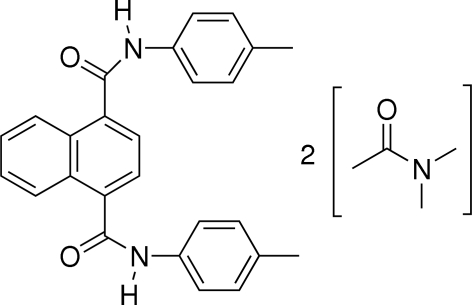


## Experimental

### 

#### Crystal data

C_26_H_22_N_2_O_2_·2C_4_H_9_NO
*M* *_r_* = 568.70
Monoclinic, 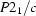

*a* = 13.270 (3) Å
*b* = 20.285 (4) Å
*c* = 12.125 (3) Å
β = 101.021 (4)°
*V* = 3203.7 (12) Å^3^
*Z* = 4
Mo *K*α radiation
μ = 0.08 mm^−1^
*T* = 294 (2) K
0.24 × 0.22 × 0.16 mm

#### Data collection

Bruker SMART CCD area-detector diffractometer
Absorption correction: multi-scan (*SADABS*; Sheldrick, 1996 ▶) *T*_min_ = 0.982, *T*_max_ = 0.988
16193 measured reflections
5652 independent reflections
2347 reflections with *I* > 2σ(*I*)
*R*_int_ = 0.057

#### Refinement

*R*[*F* ^2^ > 2σ(*F* ^2^)] = 0.055
*wR*(*F* ^2^) = 0.186
*S* = 1.00
5652 reflections
491 parameters
166 restraints
H-atom parameters constrained
Δρ_max_ = 0.20 e Å^−3^
Δρ_min_ = −0.17 e Å^−3^

### 

Data collection: *SMART* (Bruker, 1998 ▶); cell refinement: *SAINT* (Bruker, 1999 ▶); data reduction: *SAINT*; program(s) used to solve structure: *SHELXS97* (Sheldrick, 2008 ▶); program(s) used to refine structure: *SHELXL97* (Sheldrick, 2008 ▶); molecular graphics: *XP* in *SHELXTL* (Sheldrick, 2008 ▶); software used to prepare material for publication: *SHELXL97*.

## Supplementary Material

Crystal structure: contains datablocks global, I. DOI: 10.1107/S1600536808037422/ci2697sup1.cif

Structure factors: contains datablocks I. DOI: 10.1107/S1600536808037422/ci2697Isup2.hkl

Additional supplementary materials:  crystallographic information; 3D view; checkCIF report

## Figures and Tables

**Table 1:** Hydrogen-bond geometry (Å, °)

*D*—H⋯*A*	*D*—H	H⋯*A*	*D*⋯*A*	*D*—H⋯*A*
N1—H1N⋯O4	0.86	2.02	2.880 (15)	175
N2—H2N⋯O3^i^	0.86	1.99	2.821 (13)	162
C24—H24⋯O1^ii^	0.93	2.57	3.466 (5)	163
C17—H17⋯O1	0.93	2.33	2.911 (4)	120
C25—H25⋯O2	0.93	2.33	2.907 (4)	120

Symmetry codes: (i) 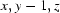; (ii) 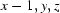.

**Fig. 1.**
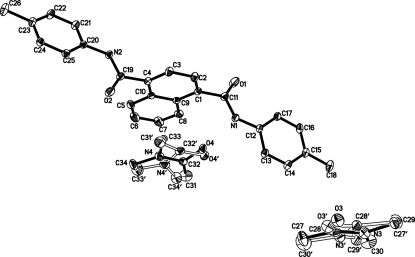
The asymmetric unit of the title compound. Displacement ellipsoids are drawn at the 30% probability level. All disordered components are shown. H atoms have been omitted for clarity.

## supplementary crystallographic information

### Comment

1,4-Naphthalenedicarboxylic acid derivatives are a class of intermediates important for applications as monomers in the preparation of polymers (Fukuzumi *et al.*, 1994; Tsukada *et al.*, 1994). Previously, we have reported the crystal structures of *N*,*N*'-bis(4-nitrophenyl)naphthalene-1,4-dicarboxamide dimethylsulfoxide disolvate (Jing *et al.*, 2006*a*) and *N*,*N*'-bis(2-methoxyphenyl)naphthalene-1,4-dicarboxamide (Jing *et al.*, 2006*b*). We now report the crystal structure of the title compound.

Bond lengths and angles in the title compound are normal. The naphthalene ring system is planar, with a maximum deviation of 0.025 (1) Å for atom C3. The two C?O gruops exhibit anti orientations. As a result of steric effects, the substituent groups at atoms C1 and C4 are twisted away from the plane of the naphthalene ring system (Fig. 1). The O1/N1/C1/C11 and O2/N2/C4/C19 planes form dihedral angles of 88.89 (1) and 89.08 (1)°, respectively, with the C1—C4/C9/C10 plane. The O1/N1/C1/C11 and C12—C17 planes are inclined at an angle of 7.48 (2)° while the O2/N2/C4/C19 and C20—C25 planes make a dihedral angle of 11.10 (2)°. The crystal packing is stabilized by N—H···O and C—H···O hydrogen bonds (Table 1).

### Experimental

Naphthalene-1,4-dicarboxylic acid (2 mmol) and an excess of thionyl chloride (6 mmol) in dioxane (20 ml) were boiled under reflux for 6 h. The solution was distilled under reduced pressure and a yellow solid was obtained. P-toluidine (4 mmol) in tetrahydrofuran (20 ml) was added to the yellow solid and boiled under reflux for 1 d. The solution was then cooled to ambient temperature and filtered to remove the tetrahydrofuran. The precipitate was dissolved in dimethylacetamide and allowed to stand for one month at ambient temperature, after which time colourless single crystals of the title compound suitable for X-ray diffraction were obtained.

### Refinement

Both *N*,*N*-dimethylacetamide molecules are disordered over two positions. The site-occupation factors for the disordered atoms were refined to 0.655 (12) and 0.345 (12), respectively, for the major and minor components of one of the dimethylacetamide molecules (with O3), and to 0.531 (13) and 0.469 (13), respectively, for the major and minor components of the other molecule. The C?O bond lengths in the major and minor components were restrained to be approximately equal. The N—C and C—C distances involving the disordered atoms were restrained to 1.48 (1) Å and 1.54 (1) Å, respectively, and the U^ij^ components were restrained to approximately isotropic behaviour. All H atoms were placed in calculated positions, with C—H = 0.93 or 0.96 Å, N—H = 0.86 Å, and refined using a riding model, with *U*_iso_(H) = 1.2*U*_eq_(C,*N*) and 1.5*U*_eq_(methyl C).

### Crystal data

**Table:**

C_26_H_22_N_2_O_2_·2C_4_H_9_NO	*F*_000_ = 1216
*M**_r_* = 568.70	*D*_x_ = 1.179 Mg m^−^^3^
Monoclinic, *P*2_1_/*c*	Mo *K*α radiation λ = 0.71073 Å
Hall symbol: -P 2ybc	Cell parameters from 2185 reflections
*a* = 13.270 (3) Å	θ = 2.3–20.1º
*b* = 20.285 (4) Å	µ = 0.08 mm^−^^1^
*c* = 12.125 (3) Å	*T* = 294 (2) K
β = 101.021 (4)º	Block, colourless
*V* = 3203.7 (12) Å^3^	0.24 × 0.22 × 0.16 mm
*Z* = 4	

### Data collection

**Table:**

Bruker SMART CCD area-detector diffractometer	5652 independent reflections
Radiation source: fine-focus sealed tube	2347 reflections with *I* > 2σ(*I*)
Monochromator: graphite	*R*_int_ = 0.057
*T* = 294(2) K	θ_max_ = 25.0º
φ and ω scans	θ_min_ = 1.6º
Absorption correction: multi-scan(SADABS; Sheldrick, 1996)	*h* = −15→13
*T*_min_ = 0.982, *T*_max_ = 0.988	*k* = −22→24
16193 measured reflections	*l* = −14→14

### Refinement

**Table:**

Refinement on *F*^2^	Secondary atom site location: difference Fourier map
Least-squares matrix: full	Hydrogen site location: inferred from neighbouring sites
*R*[*F*^2^ > 2σ(*F*^2^)] = 0.055	H-atom parameters constrained
*wR*(*F*^2^) = 0.186	*w* = 1/[σ^2^(*F*_o_^2^) + (0.0808*P*)^2^ + 0.1377*P*] where *P* = (*F*_o_^2^ + 2*F*_c_^2^)/3
*S* = 1.00	(Δ/σ)_max_ = 0.001
5652 reflections	Δρ_max_ = 0.20 e Å^−^^3^
491 parameters	Δρ_min_ = −0.17 e Å^−^^3^
166 restraints	Extinction correction: none
Primary atom site location: structure-invariant direct methods	

### Special details

**Table:**

Geometry. All e.s.d.'s (except the e.s.d. in the dihedral angle between two l.s. planes) are estimated using the full covariance matrix. The cell e.s.d.'s are taken into account individually in the estimation of e.s.d.'s in distances, angles and torsion angles; correlations between e.s.d.'s in cell parameters are only used when they are defined by crystal symmetry. An approximate (isotropic) treatment of cell e.s.d.'s is used for estimating e.s.d.'s involving l.s. planes.
Refinement. Refinement of *F*^2^ against ALL reflections. The weighted *R*-factor *wR* and goodness of fit *S* are based on *F*^2^, conventional *R*-factors *R* are based on *F*, with *F* set to zero for negative *F*^2^. The threshold expression of *F*^2^ > σ(*F*^2^) is used only for calculating *R*-factors(gt) *etc*. and is not relevant to the choice of reflections for refinement. *R*-factors based on *F*^2^ are statistically about twice as large as those based on *F*, and *R*- factors based on ALL data will be even larger.

### Fractional atomic coordinates and isotropic or equivalent isotropic displacement parameters (Å^2^)

**Table:**

	*x*	*y*	*z*	*U*_iso_*/*U*_eq_	Occ. (<1)
O1	0.4931 (2)	0.09472 (15)	0.2665 (3)	0.1430 (14)	
O2	−0.05256 (19)	0.11822 (14)	0.3003 (3)	0.1171 (11)	
N1	0.46882 (18)	0.18251 (13)	0.3719 (2)	0.0666 (8)	
H1N	0.4215	0.2032	0.3973	0.080*	
N2	−0.03759 (18)	0.02910 (13)	0.1927 (2)	0.0664 (8)	
H2N	0.0069	0.0073	0.1643	0.080*	
C1	0.3245 (2)	0.11496 (15)	0.2971 (3)	0.0594 (9)	
C2	0.2572 (3)	0.14196 (16)	0.2104 (3)	0.0777 (11)	
H2	0.2814	0.1693	0.1596	0.093*	
C3	0.1517 (3)	0.12923 (17)	0.1964 (3)	0.0765 (10)	
H3	0.1072	0.1479	0.1360	0.092*	
C4	0.1134 (2)	0.09031 (15)	0.2692 (3)	0.0591 (9)	
C5	0.1465 (3)	0.01648 (19)	0.4362 (3)	0.0829 (11)	
H5	0.0766	0.0080	0.4282	0.100*	
C6	0.2130 (4)	−0.0127 (2)	0.5205 (4)	0.1090 (15)	
H6	0.1886	−0.0409	0.5699	0.131*	
C7	0.3182 (3)	−0.0005 (2)	0.5337 (4)	0.1084 (14)	
H7	0.3633	−0.0207	0.5920	0.130*	
C8	0.3555 (3)	0.0403 (2)	0.4629 (3)	0.0825 (11)	
H8	0.4258	0.0476	0.4731	0.099*	
C9	0.2886 (2)	0.07204 (16)	0.3732 (3)	0.0592 (9)	
C10	0.1816 (2)	0.05967 (16)	0.3599 (3)	0.0593 (8)	
C11	0.4374 (3)	0.12956 (19)	0.3096 (3)	0.0738 (10)	
C12	0.5683 (2)	0.20955 (16)	0.4022 (3)	0.0609 (9)	
C13	0.5808 (3)	0.26050 (16)	0.4800 (3)	0.0708 (10)	
H13	0.5248	0.2755	0.5087	0.085*	
C14	0.6758 (3)	0.28904 (17)	0.5148 (3)	0.0814 (11)	
H14	0.6825	0.3231	0.5671	0.098*	
C15	0.7607 (3)	0.26879 (19)	0.4749 (3)	0.0771 (10)	
C16	0.7470 (3)	0.2176 (2)	0.3972 (4)	0.0871 (12)	
H16	0.8033	0.2023	0.3693	0.104*	
C17	0.6523 (3)	0.18870 (17)	0.3603 (3)	0.0778 (10)	
H17	0.6453	0.1551	0.3071	0.093*	
C18	0.8651 (3)	0.2990 (2)	0.5168 (4)	0.1091 (14)	
H18A	0.8603	0.3461	0.5098	0.164*	
H18B	0.9128	0.2827	0.4729	0.164*	
H18C	0.8884	0.2874	0.5942	0.164*	
C19	−0.0006 (2)	0.08077 (18)	0.2561 (3)	0.0666 (9)	
C20	−0.1393 (2)	0.00511 (16)	0.1657 (3)	0.0575 (8)	
C21	−0.1533 (3)	−0.05613 (17)	0.1166 (3)	0.0768 (10)	
H21	−0.0971	−0.0799	0.1029	0.092*	
C22	−0.2507 (3)	−0.08224 (18)	0.0876 (3)	0.0858 (11)	
H22	−0.2588	−0.1237	0.0545	0.103*	
C23	−0.3361 (3)	−0.0490 (2)	0.1061 (3)	0.0756 (10)	
C24	−0.3205 (2)	0.0123 (2)	0.1551 (3)	0.0714 (10)	
H24	−0.3769	0.0360	0.1688	0.086*	
C25	−0.2237 (2)	0.03961 (16)	0.1848 (3)	0.0639 (9)	
H25	−0.2156	0.0812	0.2175	0.077*	
C26	−0.4417 (3)	−0.0792 (2)	0.0734 (4)	0.1198 (16)	
H26A	−0.4894	−0.0549	0.1080	0.180*	
H26B	−0.4396	−0.1242	0.0982	0.180*	
H26C	−0.4632	−0.0776	−0.0068	0.180*	
O3	0.1070 (8)	0.9382 (7)	0.1400 (9)	0.095 (3)	0.655 (12)
N3	0.2684 (5)	0.9101 (3)	0.2237 (5)	0.081 (2)	0.655 (12)
C27	0.1348 (13)	0.8495 (10)	0.2960 (17)	0.137 (6)	0.655 (12)
H27A	0.1942	0.8346	0.3480	0.206*	0.655 (12)
H27B	0.1039	0.8130	0.2516	0.206*	0.655 (12)
H27C	0.0862	0.8680	0.3368	0.206*	0.655 (12)
C28	0.1664 (6)	0.9019 (4)	0.2194 (7)	0.083 (3)	0.655 (12)
C29	0.2879 (11)	0.9599 (10)	0.1401 (15)	0.088 (5)	0.655 (12)
H29A	0.2241	0.9793	0.1041	0.131*	0.655 (12)
H29B	0.3199	0.9390	0.0847	0.131*	0.655 (12)
H29C	0.3324	0.9937	0.1776	0.131*	0.655 (12)
C30	0.3530 (10)	0.8759 (9)	0.2965 (15)	0.126 (5)	0.655 (12)
H30A	0.3258	0.8429	0.3395	0.190*	0.655 (12)
H30B	0.3927	0.9070	0.3465	0.190*	0.655 (12)
H30C	0.3961	0.8552	0.2513	0.190*	0.655 (12)
O3'	0.0993 (13)	0.9258 (12)	0.1737 (17)	0.080 (5)	0.345 (12)
N3'	0.2233 (13)	0.8814 (6)	0.2616 (10)	0.090 (5)	0.345 (12)
C27'	0.301 (2)	0.953 (2)	0.143 (4)	0.130 (17)	0.345 (12)
H27D	0.2850	0.9856	0.0839	0.194*	0.345 (12)
H27E	0.3365	0.9170	0.1168	0.194*	0.345 (12)
H27F	0.3434	0.9730	0.2071	0.194*	0.345 (12)
C28'	0.2000 (11)	0.9283 (6)	0.1748 (10)	0.071 (5)	0.345 (12)
C29'	0.3298 (15)	0.8591 (17)	0.298 (3)	0.124 (10)	0.345 (12)
H29D	0.3735	0.8830	0.2571	0.187*	0.345 (12)
H29E	0.3342	0.8128	0.2829	0.187*	0.345 (12)
H29F	0.3515	0.8670	0.3768	0.187*	0.345 (12)
C30'	0.151 (3)	0.844 (3)	0.315 (4)	0.190 (19)	0.345 (12)
H30D	0.0819	0.8562	0.2829	0.286*	0.345 (12)
H30E	0.1641	0.8526	0.3944	0.286*	0.345 (12)
H30F	0.1598	0.7973	0.3033	0.286*	0.345 (12)
O4	0.3102 (10)	0.2454 (9)	0.4665 (12)	0.084 (3)	0.531 (13)
N4	0.1781 (6)	0.2063 (5)	0.5319 (8)	0.096 (4)	0.531 (13)
C31	0.3555 (14)	0.1799 (13)	0.6528 (16)	0.151 (7)	0.531 (13)
H31A	0.3158	0.1577	0.7001	0.227*	0.531 (13)
H31B	0.3969	0.2133	0.6954	0.227*	0.531 (13)
H31C	0.3990	0.1486	0.6253	0.227*	0.531 (13)
C32	0.2843 (6)	0.2111 (5)	0.5553 (10)	0.085 (4)	0.531 (13)
C33	0.1296 (13)	0.2400 (13)	0.4258 (17)	0.098 (8)	0.531 (13)
H33A	0.1820	0.2583	0.3904	0.147*	0.531 (13)
H33B	0.0855	0.2746	0.4426	0.147*	0.531 (13)
H33C	0.0898	0.2087	0.3761	0.147*	0.531 (13)
C34	0.1140 (10)	0.1725 (7)	0.5989 (12)	0.108 (4)	0.531 (13)
H34A	0.1568	0.1486	0.6584	0.162*	0.531 (13)
H34B	0.0690	0.1424	0.5522	0.162*	0.531 (13)
H34C	0.0740	0.2043	0.6305	0.162*	0.531 (13)
O4'	0.3332 (11)	0.2433 (12)	0.5008 (14)	0.096 (5)	0.469 (13)
N4'	0.2303 (10)	0.1904 (5)	0.5850 (9)	0.100 (4)	0.469 (13)
C31'	0.1215 (14)	0.241 (2)	0.436 (2)	0.134 (14)	0.469 (13)
H31D	0.1186	0.2660	0.3683	0.201*	0.469 (13)
H31E	0.0891	0.2653	0.4875	0.201*	0.469 (13)
H31F	0.0863	0.1998	0.4185	0.201*	0.469 (13)
C32'	0.2346 (10)	0.2275 (5)	0.4902 (9)	0.083 (4)	0.469 (13)
C33'	0.1508 (14)	0.1589 (12)	0.6330 (19)	0.181 (10)	0.469 (13)
H33D	0.0851	0.1670	0.5860	0.271*	0.469 (13)
H33E	0.1513	0.1764	0.7066	0.271*	0.469 (13)
H33F	0.1631	0.1122	0.6382	0.271*	0.469 (13)
C34'	0.3217 (13)	0.1756 (14)	0.6665 (19)	0.129 (6)	0.469 (13)
H34D	0.3804	0.1939	0.6419	0.193*	0.469 (13)
H34E	0.3295	0.1287	0.6741	0.193*	0.469 (13)
H34F	0.3161	0.1945	0.7377	0.193*	0.469 (13)

### Atomic displacement parameters (Å^2^)

**Table:**

	*U*^11^	*U*^22^	*U*^33^	*U*^12^	*U*^13^	*U*^23^
O1	0.0691 (18)	0.147 (3)	0.216 (4)	−0.0110 (18)	0.037 (2)	−0.109 (3)
O2	0.0686 (17)	0.127 (2)	0.155 (3)	−0.0018 (16)	0.0209 (17)	−0.079 (2)
N1	0.0541 (17)	0.0594 (18)	0.087 (2)	−0.0032 (14)	0.0153 (14)	−0.0136 (16)
N2	0.0448 (16)	0.0693 (19)	0.083 (2)	0.0012 (14)	0.0061 (13)	−0.0180 (16)
C1	0.054 (2)	0.054 (2)	0.067 (2)	0.0004 (16)	0.0042 (17)	−0.0066 (18)
C2	0.069 (3)	0.070 (2)	0.091 (3)	−0.006 (2)	0.008 (2)	0.019 (2)
C3	0.061 (2)	0.079 (3)	0.081 (3)	−0.0011 (19)	−0.0060 (19)	0.020 (2)
C4	0.055 (2)	0.057 (2)	0.062 (2)	−0.0039 (16)	0.0025 (17)	−0.0029 (18)
C5	0.076 (2)	0.099 (3)	0.076 (3)	−0.003 (2)	0.020 (2)	0.011 (2)
C6	0.106 (4)	0.141 (4)	0.081 (3)	0.014 (3)	0.021 (3)	0.045 (3)
C7	0.097 (4)	0.147 (4)	0.076 (3)	0.023 (3)	0.004 (3)	0.032 (3)
C8	0.067 (2)	0.110 (3)	0.065 (3)	0.015 (2)	0.001 (2)	0.002 (2)
C9	0.059 (2)	0.065 (2)	0.050 (2)	0.0054 (17)	0.0028 (16)	−0.0054 (17)
C10	0.057 (2)	0.064 (2)	0.056 (2)	0.0008 (17)	0.0071 (16)	−0.0060 (18)
C11	0.058 (2)	0.073 (3)	0.090 (3)	0.002 (2)	0.0111 (19)	−0.019 (2)
C12	0.052 (2)	0.053 (2)	0.078 (2)	−0.0033 (17)	0.0124 (17)	0.0028 (18)
C13	0.061 (2)	0.058 (2)	0.093 (3)	0.0001 (18)	0.0137 (19)	−0.006 (2)
C14	0.071 (3)	0.064 (2)	0.104 (3)	−0.010 (2)	0.004 (2)	−0.005 (2)
C15	0.059 (2)	0.070 (3)	0.098 (3)	−0.010 (2)	0.004 (2)	0.011 (2)
C16	0.060 (2)	0.088 (3)	0.118 (3)	−0.005 (2)	0.029 (2)	0.006 (3)
C17	0.065 (2)	0.074 (2)	0.098 (3)	−0.011 (2)	0.027 (2)	−0.016 (2)
C18	0.065 (3)	0.108 (3)	0.146 (4)	−0.022 (2)	0.001 (2)	0.018 (3)
C19	0.056 (2)	0.070 (2)	0.069 (2)	0.001 (2)	−0.0005 (18)	−0.007 (2)
C20	0.050 (2)	0.060 (2)	0.061 (2)	0.0030 (17)	0.0064 (15)	−0.0033 (17)
C21	0.061 (2)	0.064 (2)	0.102 (3)	0.0051 (19)	0.0074 (19)	−0.013 (2)
C22	0.071 (3)	0.066 (2)	0.115 (3)	−0.009 (2)	0.004 (2)	−0.008 (2)
C23	0.057 (2)	0.077 (3)	0.089 (3)	−0.010 (2)	0.0021 (19)	0.012 (2)
C24	0.051 (2)	0.094 (3)	0.068 (2)	0.006 (2)	0.0067 (17)	0.006 (2)
C25	0.057 (2)	0.071 (2)	0.060 (2)	0.0074 (19)	0.0031 (16)	−0.0025 (17)
C26	0.070 (3)	0.117 (3)	0.163 (4)	−0.027 (2)	−0.003 (3)	0.019 (3)
O3	0.081 (5)	0.094 (6)	0.111 (7)	0.022 (3)	0.026 (4)	0.003 (5)
N3	0.079 (4)	0.080 (4)	0.085 (4)	0.016 (3)	0.017 (3)	0.008 (3)
C27	0.157 (9)	0.145 (9)	0.126 (8)	−0.038 (6)	0.070 (6)	0.034 (6)
C28	0.086 (6)	0.081 (6)	0.085 (6)	0.013 (5)	0.024 (5)	−0.012 (4)
C29	0.105 (7)	0.084 (7)	0.086 (7)	−0.017 (5)	0.048 (5)	0.005 (4)
C30	0.120 (7)	0.116 (8)	0.127 (7)	0.042 (7)	−0.018 (6)	0.013 (6)
O3'	0.069 (7)	0.084 (8)	0.084 (9)	0.030 (6)	0.004 (6)	0.014 (6)
N3'	0.095 (9)	0.087 (8)	0.091 (8)	−0.003 (7)	0.024 (6)	0.009 (6)
C27'	0.143 (19)	0.120 (19)	0.133 (19)	−0.002 (9)	0.045 (10)	0.008 (10)
C28'	0.065 (9)	0.073 (8)	0.081 (8)	0.000 (7)	0.024 (7)	−0.006 (6)
C29'	0.119 (13)	0.113 (13)	0.127 (12)	0.005 (9)	−0.013 (8)	0.027 (9)
C30'	0.19 (2)	0.19 (2)	0.19 (2)	−0.004 (10)	0.056 (11)	0.021 (10)
O4	0.049 (5)	0.081 (5)	0.124 (8)	−0.005 (4)	0.025 (5)	−0.023 (6)
N4	0.107 (6)	0.089 (6)	0.100 (7)	−0.006 (5)	0.041 (5)	−0.014 (5)
C31	0.169 (11)	0.133 (9)	0.147 (10)	0.028 (9)	0.018 (8)	0.001 (7)
C32	0.081 (6)	0.083 (7)	0.097 (8)	−0.002 (6)	0.031 (6)	−0.029 (5)
C33	0.078 (9)	0.103 (10)	0.110 (10)	−0.010 (6)	0.010 (6)	−0.035 (7)
C34	0.117 (7)	0.109 (7)	0.115 (7)	−0.019 (6)	0.065 (6)	0.013 (6)
O4'	0.083 (8)	0.098 (7)	0.108 (8)	−0.017 (6)	0.025 (6)	−0.025 (6)
N4'	0.127 (9)	0.084 (6)	0.094 (7)	0.001 (6)	0.032 (6)	−0.014 (5)
C31'	0.126 (16)	0.145 (16)	0.136 (16)	−0.001 (9)	0.034 (9)	−0.024 (9)
C32'	0.085 (8)	0.068 (6)	0.093 (7)	0.008 (6)	0.011 (7)	−0.026 (5)
C33'	0.196 (13)	0.186 (13)	0.172 (12)	−0.016 (9)	0.063 (9)	0.015 (9)
C34'	0.121 (9)	0.127 (9)	0.139 (10)	0.017 (8)	0.025 (8)	0.014 (7)

### Geometric parameters (Å, °)

**Table:**

O1—C11	1.211 (4)	N3—C28	1.356 (7)
O2—C19	1.216 (4)	N3—C29	1.488 (10)
N1—C11	1.333 (4)	N3—C30	1.464 (8)
N1—C12	1.411 (4)	C27—C28	1.522 (9)
N1—H1N	0.86	C27—H27A	0.96
N2—C19	1.337 (4)	C27—H27B	0.96
N2—C20	1.413 (4)	C27—H27C	0.96
N2—H2N	0.86	C29—H29A	0.96
C1—C2	1.358 (4)	C29—H29B	0.96
C1—C9	1.416 (4)	C29—H29C	0.96
C1—C11	1.506 (4)	C30—H30A	0.96
C2—C3	1.401 (4)	C30—H30B	0.96
C2—H2	0.93	C30—H30C	0.96
C3—C4	1.353 (4)	O3'—C28'	1.336 (15)
C3—H3	0.93	N3'—C28'	1.409 (10)
C4—C10	1.426 (4)	N3'—C30'	1.473 (11)
C4—C19	1.504 (4)	N3'—C29'	1.469 (10)
C5—C6	1.352 (5)	C27'—C28'	1.549 (11)
C5—C10	1.416 (4)	C27'—H27D	0.96
C5—H5	0.93	C27'—H27E	0.96
C6—C7	1.397 (5)	C27'—H27F	0.96
C6—H6	0.93	C29'—H29D	0.96
C7—C8	1.351 (5)	C29'—H29E	0.96
C7—H7	0.93	C29'—H29F	0.96
C8—C9	1.421 (4)	C30'—H30D	0.96
C8—H8	0.93	C30'—H30E	0.96
C9—C10	1.420 (4)	C30'—H30F	0.96
C12—C17	1.378 (4)	O4—C32	1.379 (13)
C12—C13	1.387 (4)	N4—C32	1.388 (9)
C13—C14	1.378 (4)	N4—C34	1.454 (8)
C13—H13	0.93	N4—C33	1.490 (10)
C14—C15	1.372 (5)	C31—C32	1.505 (10)
C14—H14	0.93	C31—H31A	0.96
C15—C16	1.391 (5)	C31—H31B	0.96
C15—C18	1.511 (4)	C31—H31C	0.96
C16—C17	1.381 (4)	C33—H33A	0.96
C16—H16	0.93	C33—H33B	0.96
C17—H17	0.93	C33—H33C	0.96
C18—H18A	0.96	C34—H34A	0.96
C18—H18B	0.96	C34—H34B	0.96
C18—H18C	0.96	C34—H34C	0.96
C20—C21	1.375 (4)	O4'—C32'	1.329 (14)
C20—C25	1.378 (4)	N4'—C32'	1.384 (9)
C21—C22	1.379 (4)	N4'—C34'	1.442 (10)
C21—H21	0.93	N4'—C33'	1.447 (10)
C22—C23	1.374 (5)	C31'—C32'	1.544 (11)
C22—H22	0.93	C31'—H31D	0.96
C23—C24	1.376 (5)	C31'—H31E	0.96
C23—C26	1.511 (5)	C31'—H31F	0.96
C24—C25	1.382 (4)	C33'—H33D	0.96
C24—H24	0.93	C33'—H33E	0.96
C25—H25	0.93	C33'—H33F	0.96
C26—H26A	0.96	C34'—H34D	0.96
C26—H26B	0.96	C34'—H34E	0.96
C26—H26C	0.96	C34'—H34F	0.96
O3—C28	1.342 (11)		
			
C11—N1—C12	129.5 (3)	C28—C27—H27A	109.5
C11—N1—H1N	115.2	C28—C27—H27B	109.4
C12—N1—H1N	115.2	H27A—C27—H27B	109.5
C19—N2—C20	129.2 (3)	C28—C27—H27C	109.5
C19—N2—H2N	115.4	H27A—C27—H27C	109.5
C20—N2—H2N	115.4	H27B—C27—H27C	109.5
C2—C1—C9	119.9 (3)	O3—C28—N3	113.9 (10)
C2—C1—C11	119.5 (3)	O3—C28—C27	128.9 (11)
C9—C1—C11	120.5 (3)	N3—C28—C27	116.9 (10)
C1—C2—C3	120.9 (3)	N3—C29—H29A	109.5
C1—C2—H2	119.6	N3—C29—H29B	109.5
C3—C2—H2	119.6	H29A—C29—H29B	109.5
C4—C3—C2	121.3 (3)	N3—C29—H29C	109.4
C4—C3—H3	119.3	H29A—C29—H29C	109.5
C2—C3—H3	119.3	H29B—C29—H29C	109.5
C3—C4—C10	119.7 (3)	N3—C30—H30A	109.5
C3—C4—C19	120.1 (3)	N3—C30—H30B	109.4
C10—C4—C19	120.2 (3)	H30A—C30—H30B	109.5
C6—C5—C10	121.1 (4)	N3—C30—H30C	109.5
C6—C5—H5	119.4	H30A—C30—H30C	109.5
C10—C5—H5	119.4	H30B—C30—H30C	109.5
C5—C6—C7	120.2 (4)	C28'—N3'—C30'	128 (3)
C5—C6—H6	119.9	C28'—N3'—C29'	119.4 (19)
C7—C6—H6	119.9	C30'—N3'—C29'	112 (3)
C8—C7—C6	121.0 (4)	C28'—C27'—H27D	109.4
C8—C7—H7	119.5	C28'—C27'—H27E	109.5
C6—C7—H7	119.5	H27D—C27'—H27E	109.5
C7—C8—C9	120.9 (4)	C28'—C27'—H27F	109.6
C7—C8—H8	119.6	H27D—C27'—H27F	109.5
C9—C8—H8	119.6	H27E—C27'—H27F	109.5
C1—C9—C10	119.2 (3)	O3'—C28'—N3'	93.4 (16)
C1—C9—C8	122.6 (3)	O3'—C28'—C27'	157 (2)
C10—C9—C8	118.1 (3)	N3'—C28'—C27'	109 (2)
C5—C10—C9	118.8 (3)	N3'—C29'—H29D	109.4
C5—C10—C4	122.3 (3)	N3'—C29'—H29E	109.4
C9—C10—C4	118.9 (3)	H29D—C29'—H29E	109.5
O1—C11—N1	124.3 (3)	N3'—C29'—H29F	109.6
O1—C11—C1	121.3 (3)	H29D—C29'—H29F	109.5
N1—C11—C1	114.4 (3)	H29E—C29'—H29F	109.5
C17—C12—C13	118.6 (3)	N3'—C30'—H30D	109.5
C17—C12—N1	124.6 (3)	N3'—C30'—H30E	109.4
C13—C12—N1	116.8 (3)	H30D—C30'—H30E	109.5
C14—C13—C12	120.4 (3)	N3'—C30'—H30F	109.6
C14—C13—H13	119.8	H30D—C30'—H30F	109.5
C12—C13—H13	119.8	H30E—C30'—H30F	109.5
C15—C14—C13	122.0 (4)	C32—N4—C34	126.8 (12)
C15—C14—H14	119.0	C32—N4—C33	113.5 (12)
C13—C14—H14	119.0	C34—N4—C33	119.8 (11)
C14—C15—C16	117.0 (3)	C32—C31—H31A	109.4
C14—C15—C18	121.5 (4)	C32—C31—H31B	109.5
C16—C15—C18	121.5 (4)	H31A—C31—H31B	109.5
C17—C16—C15	121.9 (3)	C32—C31—H31C	109.5
C17—C16—H16	119.0	H31A—C31—H31C	109.5
C15—C16—H16	119.0	H31B—C31—H31C	109.5
C12—C17—C16	120.1 (3)	O4—C32—N4	105.8 (12)
C12—C17—H17	120.0	O4—C32—C31	127.8 (12)
C16—C17—H17	120.0	N4—C32—C31	126.2 (13)
C15—C18—H18A	109.5	N4—C33—H33A	109.5
C15—C18—H18B	109.5	N4—C33—H33B	109.5
H18A—C18—H18B	109.5	H33A—C33—H33B	109.5
C15—C18—H18C	109.5	N4—C33—H33C	109.5
H18A—C18—H18C	109.5	H33A—C33—H33C	109.5
H18B—C18—H18C	109.5	H33B—C33—H33C	109.5
O2—C19—N2	124.5 (3)	N4—C34—H34A	109.5
O2—C19—C4	121.1 (3)	N4—C34—H34B	109.5
N2—C19—C4	114.4 (3)	H34A—C34—H34B	109.5
C21—C20—C25	119.0 (3)	N4—C34—H34C	109.5
C21—C20—N2	117.1 (3)	H34A—C34—H34C	109.5
C25—C20—N2	123.9 (3)	H34B—C34—H34C	109.5
C20—C21—C22	120.0 (3)	C32'—N4'—C34'	121.2 (16)
C20—C21—H21	120.0	C32'—N4'—C33'	136.3 (16)
C22—C21—H21	120.0	C34'—N4'—C33'	102.4 (15)
C23—C22—C21	122.2 (4)	C32'—C31'—H31D	109.4
C23—C22—H22	118.9	C32'—C31'—H31E	109.5
C21—C22—H22	118.9	H31D—C31'—H31E	109.5
C22—C23—C24	117.0 (3)	C32'—C31'—H31F	109.5
C22—C23—C26	120.7 (4)	H31D—C31'—H31F	109.5
C24—C23—C26	122.3 (4)	H31E—C31'—H31F	109.5
C23—C24—C25	122.0 (3)	O4'—C32'—N4'	104.4 (15)
C23—C24—H24	119.0	O4'—C32'—C31'	149.1 (17)
C25—C24—H24	119.0	N4'—C32'—C31'	104.9 (16)
C20—C25—C24	119.9 (3)	N4'—C33'—H33D	109.5
C20—C25—H25	120.1	N4'—C33'—H33E	109.5
C24—C25—H25	120.1	H33D—C33'—H33E	109.5
C23—C26—H26A	109.5	N4'—C33'—H33F	109.5
C23—C26—H26B	109.5	H33D—C33'—H33F	109.5
H26A—C26—H26B	109.5	H33E—C33'—H33F	109.5
C23—C26—H26C	109.5	N4'—C34'—H34D	109.5
H26A—C26—H26C	109.5	N4'—C34'—H34E	109.5
H26B—C26—H26C	109.5	H34D—C34'—H34E	109.5
C28—N3—C29	111.1 (9)	N4'—C34'—H34F	109.5
C28—N3—C30	127.6 (10)	H34D—C34'—H34F	109.5
C29—N3—C30	121.3 (10)	H34E—C34'—H34F	109.5
			
C9—C1—C2—C3	1.6 (5)	C18—C15—C16—C17	178.5 (3)
C11—C1—C2—C3	179.9 (3)	C13—C12—C17—C16	1.2 (5)
C1—C2—C3—C4	0.7 (5)	N1—C12—C17—C16	−178.7 (3)
C2—C3—C4—C10	−2.2 (5)	C15—C16—C17—C12	−1.4 (6)
C2—C3—C4—C19	176.3 (3)	C20—N2—C19—O2	−2.2 (6)
C10—C5—C6—C7	0.0 (6)	C20—N2—C19—C4	178.2 (3)
C5—C6—C7—C8	−0.1 (7)	C3—C4—C19—O2	−88.3 (4)
C6—C7—C8—C9	0.2 (7)	C10—C4—C19—O2	90.2 (4)
C2—C1—C9—C10	−2.2 (5)	C3—C4—C19—N2	91.3 (4)
C11—C1—C9—C10	179.4 (3)	C10—C4—C19—N2	−90.2 (4)
C2—C1—C9—C8	177.5 (3)	C19—N2—C20—C21	−167.9 (3)
C11—C1—C9—C8	−0.8 (5)	C19—N2—C20—C25	12.8 (5)
C7—C8—C9—C1	−179.9 (4)	C25—C20—C21—C22	−0.3 (5)
C7—C8—C9—C10	−0.1 (5)	N2—C20—C21—C22	−179.7 (3)
C6—C5—C10—C9	0.0 (5)	C20—C21—C22—C23	0.0 (6)
C6—C5—C10—C4	179.0 (4)	C21—C22—C23—C24	0.1 (6)
C1—C9—C10—C5	179.8 (3)	C21—C22—C23—C26	−179.9 (4)
C8—C9—C10—C5	0.0 (4)	C22—C23—C24—C25	0.0 (5)
C1—C9—C10—C4	0.7 (4)	C26—C23—C24—C25	−179.9 (3)
C8—C9—C10—C4	−179.0 (3)	C21—C20—C25—C24	0.4 (5)
C3—C4—C10—C5	−177.6 (3)	N2—C20—C25—C24	179.8 (3)
C19—C4—C10—C5	3.9 (5)	C23—C24—C25—C20	−0.3 (5)
C3—C4—C10—C9	1.4 (5)	C29—N3—C28—O3	1.9 (13)
C19—C4—C10—C9	−177.1 (3)	C30—N3—C28—O3	−177.0 (13)
C12—N1—C11—O1	−0.4 (6)	C29—N3—C28—C27	176.7 (15)
C12—N1—C11—C1	178.8 (3)	C30—N3—C28—C27	−2.2 (17)
C2—C1—C11—O1	−90.8 (5)	C30'—N3'—C28'—O3'	5(4)
C9—C1—C11—O1	87.5 (5)	C29'—N3'—C28'—O3'	176 (2)
C2—C1—C11—N1	89.9 (4)	C30'—N3'—C28'—C27'	−178 (4)
C9—C1—C11—N1	−91.8 (4)	C29'—N3'—C28'—C27'	−6(3)
C11—N1—C12—C17	7.8 (5)	C34—N4—C32—O4	177.9 (12)
C11—N1—C12—C13	−172.1 (3)	C33—N4—C32—O4	−1.9 (17)
C17—C12—C13—C14	−0.5 (5)	C34—N4—C32—C31	3(2)
N1—C12—C13—C14	179.3 (3)	C33—N4—C32—C31	−177 (2)
C12—C13—C14—C15	0.1 (5)	C34'—N4'—C32'—O4'	−2(2)
C13—C14—C15—C16	−0.3 (5)	C33'—N4'—C32'—O4'	177 (2)
C13—C14—C15—C18	−177.9 (3)	C34'—N4'—C32'—C31'	−172 (2)
C14—C15—C16—C17	0.9 (5)	C33'—N4'—C32'—C31'	7(3)

### Hydrogen-bond geometry (Å, °)

**Table:**

*D*—H···*A*	*D*—H	H···*A*	*D*···*A*	*D*—H···*A*
N1—H1N···O4	0.86	2.02	2.880 (15)	175
N2—H2N···O3^i^	0.86	1.99	2.821 (13)	162
C24—H24···O1^ii^	0.93	2.57	3.466 (5)	163
C17—H17···O1	0.93	2.33	2.911 (4)	120
C25—H25···O2	0.93	2.33	2.907 (4)	120

Symmetry codes: (i) *x*, *y*−1, *z*; (ii) *x*−1, *y*, *z*.
